# External validation of multivariable prediction models: a systematic review of methodological conduct and reporting

**DOI:** 10.1186/1471-2288-14-40

**Published:** 2014-03-19

**Authors:** Gary S Collins, Joris A de Groot, Susan Dutton, Omar Omar, Milensu Shanyinde, Abdelouahid Tajar, Merryn Voysey, Rose Wharton, Ly-Mee Yu, Karel G Moons, Douglas G Altman

**Affiliations:** 1 Centre for Statistics in Medicine, Botnar Research Centre, University of Oxford, Windmill Road, Oxford OX3 7LD, UK; 2 Julius Center for Health Sciences and Primary Care, UMC Utrecht, Utrecht, The Netherlands

## Abstract

**Background:**

Before considering whether to use a multivariable (diagnostic or prognostic) prediction model, it is essential that its performance be evaluated in data that were not used to develop the model (referred to as external validation). We critically appraised the methodological conduct and reporting of external validation studies of multivariable prediction models.

**Methods:**

We conducted a systematic review of articles describing some form of external validation of one or more multivariable prediction models indexed in PubMed core clinical journals published in 2010. Study data were extracted in duplicate on design, sample size, handling of missing data, reference to the original study developing the prediction models and predictive performance measures.

**Results:**

11,826 articles were identified and 78 were included for full review, which described the evaluation of 120 prediction models. in participant data that were not used to develop the model. Thirty-three articles described both the development of a prediction model and an evaluation of its performance on a separate dataset, and 45 articles described only the evaluation of an existing published prediction model on another dataset. Fifty-seven percent of the prediction models were presented and evaluated as simplified scoring systems. Sixteen percent of articles failed to report the number of outcome events in the validation datasets. Fifty-four percent of studies made no explicit mention of missing data. Sixty-seven percent did not report evaluating model calibration whilst most studies evaluated model discrimination. It was often unclear whether the reported performance measures were for the full regression model or for the simplified models.

**Conclusions:**

The vast majority of studies describing some form of external validation of a multivariable prediction model were poorly reported with key details frequently not presented. The validation studies were characterised by poor design, inappropriate handling and acknowledgement of missing data and one of the most key performance measures of prediction models i.e. calibration often omitted from the publication. It may therefore not be surprising that an overwhelming majority of developed prediction models are not used in practice, when there is a dearth of well-conducted and clearly reported (external validation) studies describing their performance on independent participant data.

## Background

Prediction models are used to estimate the probability of presence of a particular disease (diagnosis) or to estimate the probability of developing a particular outcome in the future (prognosis). Published in ever increasing numbers, prediction models are now being developed in virtually all medical domains and settings [1-3]. Driving the growing number of published prediction models is the mounting awareness of the need to have accurate and objective approaches to combine multiple pieces of information (e.g. patient and disease characteristics, symptoms, test results, etc.) for an individual to derive a single estimate of risk. This is illustrated by their increasing inclusion in clinical guidelines and recommendation by national bodies [4-6]. Whilst they are not intended to replace clinical judgement, prediction models have a clear role in augmenting clinical judgement. Studies have shown prediction models provide more accurate and less variable estimates of risk compared to more subjectively made predictions [7,8]. However, whilst there is an increased awareness of the importance of prediction models, the majority of published prediction models are opportunistic and are rarely being used or even mentioned in clinical guidelines [9]. This clearly points to considerable waste in research (including monetary and scientific) [10].

Before considering whether to use a clinical prediction model, it is essential that its predictive performance be empirically evaluated in datasets that were not used to develop the model [11-13]. This is often referred to as external validation [13,14]. Performance is typically characterised by evaluating a model’s *calibration* and *discrimination*[15]. Calibration is the agreement between predicted and observed risks, whilst discrimination is the ability of the model to differentiate between patients with different outcomes [14]. Reasons for assessing performance in other datasets include quantifying optimism from model overfitting or deficiencies in the statistical modelling during model development (e.g. small sample size, inappropriate handling of missing data) and evaluating the transportability of the model in different locations consisting of plausibly similar individuals (different case-mix). External validation is exploring genuine differences in characteristics of the cohorts (between the development and validation cohorts) and examining how well the models performs. A clear distinction should also be made between estimating a model’s external performance done by the authors who developed the prediction model and done by independent investigators [16], thereby reducing inflated findings and spin [17,18]. Replicating findings obtained during the original development of the prediction model in different data but from the same underlying target population is key [19-21].

A large number of prediction models are being developed, but only a small fraction of these ever get evaluated on its performance in other participant data. Systematic reviews evaluating the methodological conduct and reporting of studies developing prediction models all conclude that these studies are characterised by deficiencies in study design, inadequate statistical methodology, and poor reporting [1,22-24]. Ultimately one is interested in how well the prediction model performs in other participants and thus well conducted and clearly reported external validation studies are essential to judge the prediction model. However, we are not aware of any systematic reviews specifically evaluating the methodological conduct and reporting of external validation studies.

The aim of this article is therefore to report a review of the methodological conduct and reporting of published articles describing the external validation of prediction models. In particular we focus on the design (including sample size), assessment of predictive performance and the quality of reporting.

## Methods

### Literature search

PubMed was searched on 02-February-2011 using the search string described in Additional file 1 to identify English-language articles that evaluated the performance of one or more multivariable clinical prediction models. Searches included articles published in 2010 belonging to the subset of 119 PubMed journals listed in Abridged Index Medicus (http://www.nlm.nih.gov/bsd/aim.html). One reviewer (GSC) examined the titles and abstracts of all articles identified by the search string to exclude articles not pertaining to clinical prediction models. Information on how the prediction models were developed is important to place the evaluation of the model in context. Therefore, for studies where the development of the model was described in a previous publication, this article was identified and retrieved, but only if this was cited in the external validation article. We took this approach as often there are multiple models known by a single name (e.g. Framingham Risk Score), multiple models for the same or similar outcome developed by the same authors and models get updated or refined. Therefore a clear reference to the article describing the development of the prediction was essential.

### Inclusion criteria

We focused our review on studies that described some form of evaluation of a multivariable prediction model, diagnostic or prognostic, and in data that were not used to develop the model. We included studies that both developed a prediction model and subsequently evaluated it on separate data, as well as studies that only described the evaluation (validation) of one or more existing prediction models in other participant data. We excluded articles where authors randomly split a single dataset into a development and validation dataset, as this does not constitute an external validation and is a weak and inefficient design [12,25]. However, studies that carried out a temporal or geographical (i.e. non-random) split were eligible and included as they are considered a particular type of external validation [13,26].

### Data extraction, analysis and reporting

Information was extracted that described aspects of model development and evaluation. Regarding the development of the model, items extracted for this review include aspects of study design (including dates of data collection), sample size (and number of events), number of predictors considered and included in the final model, whether ranges of any continuous predictors were reported, handling and reporting of missing data, type of model (including if they developed a simplified model), whether there was sufficient information to implement the model and any performance data of the prediction model. Regarding the evaluation of the model on separate data, we extracted aspects of study design (including dates of data collection), sample size (and number of events), whether any predictors or outcome were defined differently, type of model being evaluated (i.e. regression equation or a simplified model), handling and reporting of missing data and the performance measures calculated (e.g. calibration and discrimination). Items were recorded by duplicate data extraction by nine reviewers independently (AT, GSC, JdG, LMY, MS, MV, OO, RW, SD), with one reviewer (GSC) extracting information on all articles. Any disagreements were resolved by a third reviewer.

The data extraction form for this review was based largely on previous systemic reviews of studies describing the development of multivariable prediction models [1,2,22,23,27] and can be found in Additional file 2. For the primary analysis, we calculated the proportion of studies and the proportion of risk prediction models for each of the items extracted, where appropriate. To aid the interpretation of our findings, we precede each section in the results with a brief explanation of its importance.

## Results

**Figure 1:**
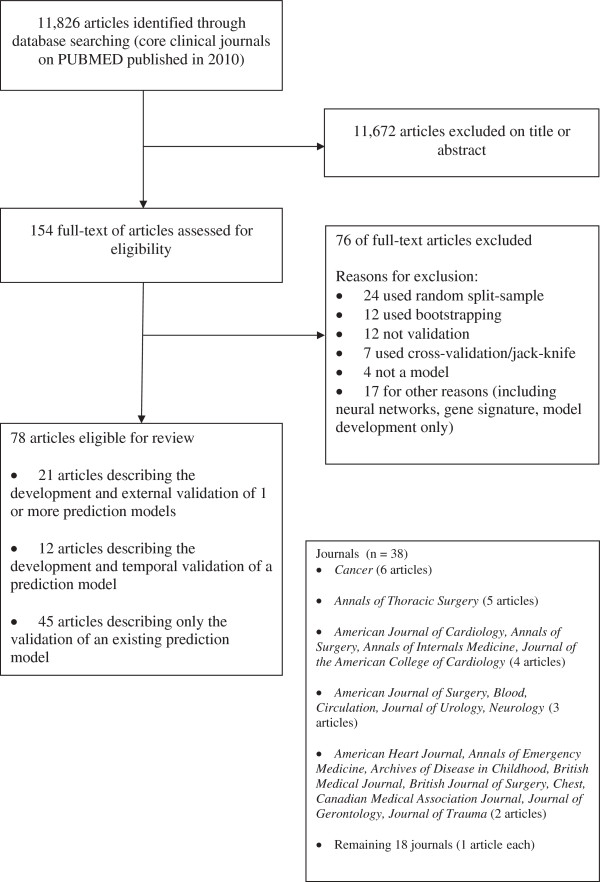
Flow of included studies.

The search string retrieved 11,826 articles in PubMed, of which 11,672 were excluded on the title or abstract. The full text of 154 eligible articles was obtained, from which 76 were excluded leaving 78 eligible for full review (Figure 1). Twenty-one articles (21/78; 27%; 95% CI 18% to 38%) had the term *‘validation’* or *‘validity’* in the title of the article, whilst four articles used the term *‘external validation’* in the title. Only one article indicated in the title that it was an external validation carried out by independent researchers. The 78 eligible articles [A1-A78] came from 37 of the core clinical journals, see Figure 1 for a breakdown of journals. Reference numbers are preceded by an A to indicate they correspond to the reference list in Additional file 3.

These 78 studies externally evaluated the performance of 120 prediction models on different data to that used in their development. The median number of predictors in the model was 6 (range 2 to 1096). Nineteen articles (19/78; 24%; 95% CI 16% to 36%) described a diagnostic prediction model, whilst 59 articles (59/78; 76%; 95% CI 64% to 84%) described a prognostic model. Most articles were published in the field of oncology (22/78; 28%; 95% CI 19% to 40%), followed by cardiovascular diseases (18/78; 23%; 95% CI 15% to 34%), see Table 1.

**Table 1:** Summary overview of included articles^*^

**Study aim**	**Cardiovascular (n = 18)**	**Oncology (n = 22)**	**Other (n = 38)**	**Aim of prediction model**		**Total articles (n = 78)**	**Number of models (n = 120)**
				**Diagnostic (n = 19)**	**Prognostic (n = 59)**		
Model development with temporal-split validation	3 (4)	1 (1)	8 (10)	2 (3)	10 (13)	12 (15)	14 (12)
Model development with external validation	7 (9)	6 (8)	8 (10)	3 (4)	18 (23)	21 (27)	38 (32)
External validation only	8 (10)	15 (19)	22 (28)	14 (18)	31 (40)	45 (58)	68 (57)

^*^Percentages are in given parentheses.

Forty-five articles (45/78; 58% 95% CI to 46% to 69%) described the evaluation (only) of 67 existing published prediction models (Table 1). Of these, 30 evaluated only a single model, whilst ten studies evaluated two models, four studies evaluated three models, and one study evaluated five prediction models. Eighteen validation only articles (18/45; 40%; 95% CI 26% to 56%) included at least one author who was also an author on the paper that developed the model being evaluated. Sixty models (60/120; 50%; 95% CI 41% to 59%) were developed using logistic regression, 32 using Cox regression (32/120; 27%; 95% CI 19% to 36%); 8 using other statistical methods (8/120; 7%; 95% CI 3% to 13%), whilst either no formal statistical modelling (including consensus approaches to select predictors and their weights) was used, no reference to the development publication or it was unclear for 20 models (20/120; 17%; 95% CI 11% to 25%). The median sample size used to develop the predictions models was 1360 with a median of 189 outcome events.

Thirty-three articles (33/78; 42%; 95% CI 31% to 54%) described both the development of a prediction model and an evaluation of its performance on a separate dataset. Twelve of these studies (12/33; 36%; 95% CI 21% to 55%) used data from the same centre but from a different time-period (temporal validation). Twenty-six of these studies (26/33; 79%; 95% CI 61% to 90%) did not compare the new model to an existing model.

### Model development: continuous predictors

Applying a prediction model to individuals whose distributions of characteristics or measurements (e.g. predictors and test results) outside the range of those used in model development is a form of extrapolation and may compromise a model’s performance. It is therefore important for authors to clearly report all ranges and categories for predictors included in the prediction model to understand a potential decrease or increase in model performance. Reporting means and standard deviations or interquartile ranges, whilst descriptive, does not indicate in whom the model is primarily applicable. For example, when a prediction model developed in participants aged 30 to 60 years is evaluated in participants aged 50 to 80 years, this should be fully acknowledged. For those using a prediction model, it is important to understand the population in whom the model was developed and in whom the model has been validated.

The ranges of any continuous predictors were only reported in the development of 10 of the models (10/120; 8%; 95% CI 4% to 15%) evaluated in the 78 articles.

### Model presentation (development) & evaluation (validation)

Evaluating the performance of a prediction model in other individuals requires making predictions for each individual from the prediction model. Whilst prediction models are generally developed using regression modelling techniques, they are often presented in a simplified format. For example, the regression coefficients for each predictor in the model are often rounded to integers, which are then summed to produce a score. For a correct evaluation of performance of these simplified models, notably a model’s calibration, providing a mechanism that relates this integer score to an absolute risk is required. Prediction models are also often presented as nomograms, which are a graphical representation; they are not a simplification. However, to efficiently evaluate the performance of the nomogram, the underlying regression model is required (and be published in the development study), as clearly using the actual nomogram (for hand calculations) is fraught with potential problems (e.g. transcription, and rounding) when used on a large number of individuals.

Sixty-two of the models evaluated (62/120; 52%; 95% CI 42% to 61%) were presented in the original development articles as simplified scoring systems (i.e. regression coefficients rounded to integers or counting risk factors) and 42 as regression models (42/120; 35%; 95% CI 27% to 44%). Ten models (10/120; 8%; 95% CI 4% to 15%) were presented as nomograms (9/10 in the field of oncology), whilst the remaining were presented as regression trees or links to a web calculator. Only nine (9/62; 15%; 95% CI 7% to 26%) scoring systems (i.e. those that had been simplified to an integer scoring system) presented a way to equate the overall integer score from the model to a predicted risk; 6 presented predicted risks for each of the integer scores in a lookup table, whilst 3 models presented this information in a plot.

The 10 nomograms were evaluated in four articles that described both a development and external validation and in six external validation only studies. Three of the six external validation studies were based on published nomograms where the underlying regression model was not reported in the original publication (only a link to a web calculator). The other three external validation studies included authors who were also authors of the original publication developing the nomogram (thus having access to the underlying regression model).

### Model validation: study design

Details on study design are key pieces of information to judge the adequacy of a model’s external validation. This includes knowing dates for the period in which study participants were recruited, to place the study in a historical context, particularly in relation to the period when the prediction model was developed. Also and more importantly, it is essential to know details regarding number of participants and in particular the number of outcome events, which is the effective sample size [1,28].

Nine studies (9/78; 12% 95% CI 6% to 21%) failed to report study dates for when the data were collected. 16 articles (16/78; 21% 95% CI 13% to 31%) failed to report the number of events in the validation datasets, see Table 2. Six studies reported only the proportion of events. One study did not report the sample size. The median sample size was 795 (range 49 to 1,117,123). For studies that reported the number of events, the median number of events was 106 (range 6 to 42,408). Forty-eight percent of datasets used to evaluate the prediction models had less than a previously recommended minimum of 100 events [28]. Seventeen studies (17/78; 22%) presented flow diagrams to describe how individuals were included.

**Table 2:** **Sample size**^**†**^

	**Development & validation articles (n = 33)**		**Validation only**
	**Development**	**Validation**	**(n = 45)**
**Sample size**			
*Explanation of sample size reported*	Information not extracted	2 (6)	4 (9)
*Articles where the number of participants were reported*	33 (100)	32 (97)	44 (98)
*Median number of participants (range)*	1360 (68, 17589824)	1041 (87, 1117123)	694 (49, 797373)
*Articles where the number of events were reported*	28 (85)	26 (79)	36 (80)
*Median number of events (range)*	189 (12, 90324)	100 (14, 3623)	108 (6, 42408)

^†^Percentages are in given parentheses.

### Model validation: handling of missing data

Missing data is common in all types of medical research, including prediction modelling studies [1,22,29]. Omitting individuals with missing data, and conducting a so-called complete-case analysis not only reduces sample size but can also lead to invalid results. Of particular concern is if those omitted are not representative of the whole population, that is the reason for the missingness is not completely at random [30]. It is therefore important to know whether individuals were omitted, and how many were omitted. If those with missing values were retained in the analyses, then it is important for the reader to know how they were handled in the analysis, including whether methods such as multiple imputation were used [31].

Table 3 describes how missing data were handled. Forty-two studies (42/78; 54%; 95% CI 42% to 65%) made no explicit mention of missing data. Fifty studies (50/78; 64%) either explicitly or implicitly (in the absence of indicating otherwise) conducted complete-case analyses. Twenty-three studies (23/78; 29%; 95% CI 20% to 41%) reported the number of individuals with missing data; 18 validation only studies and 5 combined development and validation studies. Only 8 studies (8/78; 10%; 95% CI 5% to 20%) reported the number of missing values per predictor. Seven studies used multiple imputation to replace missing values. One study that had no information recorded for one predictor imputed a value of zero for all individuals.

**Table 3:** **Handling of missing data**^**‡**^

	**Single development & validation articles**^**§ **^**(n = 33)**		**Separate development & validation articles**	
	**Development cohort**	**Validation cohort**	**Development paper**	Validation paper^**^
			**(n = 66)**	**(n = 45)**
Studies with no mention of missing data	13 (39)	21 (64)	30 (45)	21 (47)
Studies reporting number of participants with missing data	Information not extracted	5 (15)	Information not extracted	18 (40)
Studies reporting number of missing values for each predictor	Information not extracted	3 (9)	Information not extracted	5 (11)
Studies carrying out complete-case analysis^††^	26 (79)	30 (91)	43 (65)	20 (44)
Studies explicitly mentioning carrying out multiple imputation	Information not extracted	2 (6)	Information not extracted	7 (16)

^‡^Percentages are in given parentheses.

^§^Articles that developed a new model and also evaluated the performance on a separate dataset.

^**^Articles that only described the evaluation of a previously published prediction model.

^††^In the absence of clear reporting, those studies that did not mention how missing data were handled were assumed to have conducted a complete-case analysis.

### Model validation: outcome definition

The outcome to be predicted in an external validation study may be defined differently from how it was defined in the original publication describing the development of the prediction model. The outcome definition may be intentionally different (e.g. diabetes determined from using a oral glucose tolerance test or self-report [32]). Similarly, a model developed to predict an outcome at one particular time point may be evaluated to see if it is also predictive at a different time point [33].

Seventeen of the 45 validation only studies (17/45; 38%; 95% CI 24% to 53%) evaluated the performance of prediction models for outcomes (intentionally) defined differently from the original outcome definition. In six validation only studies (6/45; 13%; 95% CI 6% to 27%) it was unclear whether the definition of the outcome was the same as the original outcome definition.

### Reference to the original prediction model

Seven of the 45 validation only studies (7/45; 16%; 95% CI 7% to 30%) did not cite the original article that described the development of any of the prediction models evaluated; including one study that cited a non-existent article, cited as in-press, but has to date not been published.

### Comparison of case-mix

Thirty-one of the 78 studies (31/78; 40%; 95% CI 29% to 51%) compared or discussed the characteristics of both the development and validation cohorts. Nine of the validation only studies (9/45; 20%; 95% CI 10% to 35%) compared (either numerically or descriptively) the characteristics of the development and validation cohorts.

### Model validation: model performance measures

The two key components characterising the performance of a prediction model are calibration and discrimination [14,15,34]. Calibration is the agreement between prediction from the model and observed outcomes and reflects the predictive accuracy of the model. Discrimination refers to the ability of the prediction model to separate individuals with and without the outcome event; those with the outcome event should have a higher predicted risk compared to those who do not have the outcome event.

**Figure 2:**
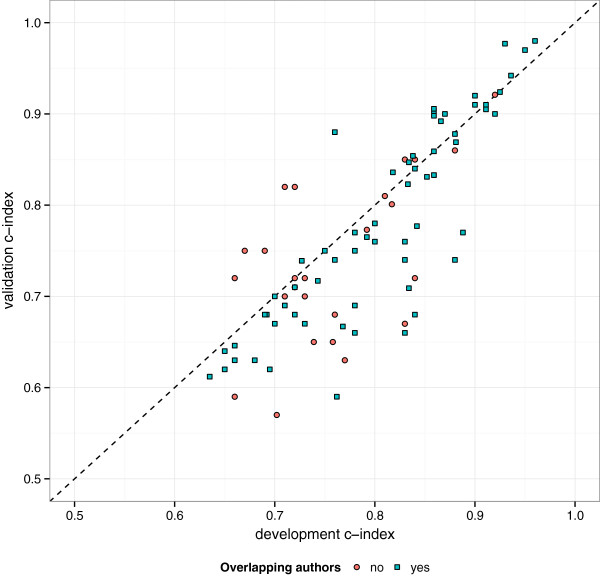
Prediction model discrimination (c-index) from the development and external validation.

Table 4 describes how the performance of the prediction models was evaluated. Fifty-three articles (53/78; 68%; 95% CI 56% to 78%) did not report evaluating a prediction model’s calibration, which can (arguably) be considered as the key performance measure of a prediction model. Fifteen studies (15/78; 21%; 95% CI 12% to 30%) calculated the Hosmer-Lemeshow goodness-of-fit test, and only 11 studies (11/78; 14% 95% CI 8% to 24%) presented a calibration plot. It was often unclear whether the reported performance measures were for the full regression model or for the simplified models, and therefore this could not be evaluated further. Fifty-seven articles (57/78; 73%; 95% CI 62% to 82%) reported an evaluation of model discrimination (e.g. *c*-index). Of these 57 articles, 17 (17/57; 30%; 95% CI 19% to 44%) did not report confidence intervals. The mean validation *c*-index in studies conducted by authors who also developed the prediction model (either in the same paper which developed the model or a subsequent external validation) was 0.78 (IQR 0.69, 0.88) compared to 0.72 (IQR 0.66, 0.77) in external validation studies carried out by independent investigators, see Figure 2.

**Table 4:** Model performance measures reported in the 78 studies

**Performance measure**	**n (%)**
**Calibration**	
*Not assessed*	53 (68)
*Calibration plot*	11 (14)
*Hosmer-Lemeshow test*^*7*^	16 (21)
**Discrimination**	
*Not assessed/not reported*	21 (27)
*c-index*	57 (73)
**ROC curve**	23 (29)
**Overall performance measures**	
*Brier score*	5 (6)
*R*^*2*^	13 (17)
**Clinical utility** (e.g. decision curve analysis)	1 (1)

^7^Including one study calculating Grønnesby and Borgan goodness-of-fit test (for survival data).

Twenty-three articles (23/78; 29%; 95% CI 20% to 41%) presented Receiver Operating Characteristic (ROC) curves, yet only four articles labelled the curve at specific points enabling sensitivity and specificity to be read off at these points.

## Discussion

We believe this is the first study that has systematically appraised the methodological conduct and reporting of studies evaluating the performance of multivariable prediction models (diagnostic and prognostic). Evaluating the performance of a prediction model in datasets not used in the derivation of the prediction model (external validation) is an invaluable and crucial step in the introduction of a new prediction model before it should be considered for routine clinical practice [12,13,26,35]. External or independent evaluation is predicated on the full reporting of the prediction model in the article describing its development, including reporting eligibility criteria (i.e. ranges of continuous predictors, such as age). A good example of a prediction model that has been inadequately reported, making evaluations by independent investigators impossible [36,37], yet appears in numerous clinical guidelines [4,38] is the FRAX model for predicting the risk of osteoporotic fracture [39].

We assessed the methodological conduct and reporting of studies published in the 119 core clinical journals listed in Abridged Index Medicus. Our review identified that 40% of external validation studies were reported in the same article that described the development of the prediction model. Of the 60% of articles that were solely evaluating the performance of an existing published prediction model, 40% were conducted by authors involved in the development of the model. Whilst evaluating one’s own prediction model is a useful first step, this is less desirable then an independent evaluation conducted by authors not involved in its development. Authors evaluating the performance of their own model are naturally likely to err on being overly optimistic in interpreting results or selective reporting (possibly selectively choosing to publish external validation from datasets with good performance and omitting any poorly performing data).

The quality of reporting in external validation studies included in this review was unsurprisingly, very poor. Important details needed to objectively judge the quality of the study were generally inadequately reported or not reported at all. Little attention was given to sample size. Whilst formal sample size calculations for external validation studies are not necessary, there was little acknowledgement that the number of events is the effective sample size; 46% of datasets had fewer than 100 events, which is indicated, though from a single simulation study, as a minimum effective sample size for external validation [28]. Around half of the studies made no explicit mention of missing data. The majority (64%) of studies were assumed to have conducted complete-case analyses to handle missing values, despite methodological guidance to do the contrary [40-44]. Multiple imputation was conducted and reported in very few studies and the amount and reasons for any missing data were poorly described. The analyses of many of these studies were often confusingly reported and conducted, with numerous unclear and unnecessary analyses done as well as key analyses (e.g. calibration) not carried out. Some aspects identified in this review are not specific to prediction modelling studies (e.g. sample size, study design, dates), it is therefore disappointing that key basic details on study are also often poorly reported.

Key characteristics, such as calibration and discrimination, are widely recommended aspects to evaluate [9,12-15,26,45,46]. Both components are extremely important and should be reported for all studies evaluating the performance of a prediction model, yet calibration, which assesses how close the prediction for an individual is to their true risk, is inexplicably rarely reported, as observed in this and other reviews [1,23,47]. With regards to calibration, preference should be to present a calibration plot, possibly with the calibration slope and intercept in rather than the Hosmer-Lemeshow test, which has a number of known weaknesses related to sample size [48]. For example a model evaluate on a large dataset with good calibration can fail the Hosmer-Lemeshow test, whilst a model validated on a small dataset with poor calibration can pass the Hosmer-Lemeshow test. Arguably, more important than calibration or discrimination, is clinical usefulness. Whilst a formal evaluation of clinical usefulness in terms of improving patients outcomes or changing clinician behavior [26,49] are not part of external validation, indicating the potential clinical utility can be determined. New methods based on decision curve analysis (net benefit) [50] and relative utility [51] have recently been introduced. Only one study in our review attempted to evaluate impact on using a model [52], which included an author who developed the particular methodology [50]. However, since this review, interest and uptake of these methods have slowly started to increase. In instances where the validation is seeking to evaluate the clinical utility, issues such as calibration (which can often be observed in a decision curve analysis) may not be necessary. However, most studies in our review were attempting to evaluate the statistical properties and thus as a minimum, we expect calibration and discrimination to be reported.

Many of the prediction models were developed and presented as simplified scoring systems, whereby the regression coefficients were rounded to integers and then summed to obtain an overall integer score for a particular individual. These scores are often then used to create risk groups, by partitioning the score into 2 or more groups. However, these groups are often merely labelled low, medium or high risk groups (in the case of 3 groups), with no indication to how low, medium or high was quantified. Occasionally, these risk groups may be described by reporting the observed risk for each group, however, these risk groups should be labelled with the predicted risks, by typically reporting the range or mean predicted risk. Authors of a few of the scoring systems presented lookup tables or plots which directly translated the total integer score to a predicted risk, making the model much more useable.

Terminology surrounding prediction modelling studies is inconsistent and identifying these studies is difficult. Search strings developed to identify prediction modelling studies [53-55] inevitably result in a large number of false-positives, as demonstrated in this review. For example, whilst the term *validation* may be semantically debatable [13], it is synonymous in prediction modelling studies as referring to evaluating performance, yet, in the studies included in this review, only 43 papers (55%) included the term in the abstract or title (24% in the title alone). To improve the retrieval of these studies we recommend authors to clearly state in the title if the article describes the development or validation (or both) of a prediction model.

Our study has the limitation that we only examined articles published in the subset of PubMed core clinical journals. We chose to examine this subset of journals as it included the 119 of the most widely read journals published in English, covering all specialties of clinical medicine and public-health sciences, and including all major medical journals. Our review also included studies published in 2010, yet since no initiative to improve the quality of reporting of prediction modelling studies has been put in place, we feel, that whilst methodology may have evolved there is no belief that reporting will have improved.

Systematic reviews of studies developing prediction models have identified numerous models for predicting the same or similar outcome [1,56-59]. Instead of developing yet another new prediction model for which several already exist, authors should direct their efforts in evaluating and comparing existing models and where necessary update or recalibrate, rather than disregard and ultimately waste information from existing studies. Journal editors and peer reviewers can also play a role by demanding clear rationale and evidence for the need of a new prediction model and place more emphasis on studies evaluating prediction models. Recently, developments have been made that combine existing prediction models, thereby improving the generalisability, but importantly not wasting existing research [60,61].

## Conclusions

The conclusions from this systematic review are consistent with those of similar reviews that have appraised the methodological conduct and quality of reporting published studies describing the development of multivariable prediction models [1,2,22,23,27]. The focus on prediction modelling studies has tended to be on how prediction models were developed, yet this is undeniably of secondary importance to assessing predictive accuracy of a model on participant data. Nonetheless, despite the obvious importance of evaluating prediction models on other datasets, this practice is relatively rare and for the majority of published validation studies, the methodology quality and reporting is worryingly poor.

Currently no reporting guidelines exist to assist authors, editors and reviewers to ensure that key details on how a prediction model has been developed and validated are clearly reported to enable readers to make an objective judgment of the study and the prediction model. A recent initiative, called TRIPOD (Transparent Reporting of a multivariable model for Individual Prognosis Or Diagnosis), will soon publish a consensus statement (along with an Explanatory document) on the minimal details to report when developing or validating a multivariable diagnostic or prognostic prediction model [62]. This initiative if adopted by journals publishing prediction modelling studies will hopefully raise the reporting standards. The results from this systematic review, will therefore also act as a baseline to compare against after the implementation of the TRIPOD guidelines.

## Competing interests

The authors declare that they have no competing interests.

## Authors’ contributions

GSC conceived the study, DGA advised on the design of the study and contributed to the protocol. GSC, JAdG, SD, OO, MS, AT, MV, RW and LMY undertook data extraction. GSC conducted the analyses of the data. All authors had full access to all the data. GSC took primary responsibility for writing the manuscript. All authors provided feedback on all versions of the paper. All authors read and approved the final manuscript.

## Pre-publication history

The pre-publication history for this paper can be accessed here:

http://www.biomedcentral.com/1471-2288/14/40/prepub

## Supplementary Material

Additional file 1: Table S1 Search string and search results (02-February-2011). Click here for file

Additional file 2: Table S2 Data Extraction Sheet. Click here for file

Additional file 3: Table S3 List of included studies. Click here for file
