# Supplementary material for: External validation of multivariable prediction models: a systematic review of methodological conduct and reporting
Source: BMC Med Res Methodol. 2014 Mar 19;14:40. doi: 10.1186/1471-2288-14-40 (PMC3999945; doi:10.1186/1471-2288-14-40)
Supplement: Additional file 1: Table S1 — Search string and search results (02-February-2011). [file 1471-2288-14-40-S1.doc]

Additional file 1: Table S1: Search string and search results (02-February-2011)

| **String No.** | **Searches** | **Count** |
| --- | --- | --- |
| 1 | (validat$ OR rule$).tw. OR predict$.ti. | 337259 |
| 2 | (Predict$ AND (Outcome$ OR Risk$ OR Model$)).tw. | 294535 |
| 3 | (History OR Variable$ OR Criteria OR Scor$ OR Characteristic$ OR Finding$ OR Factor$).tw. AND (Predict$ OR Model$ OR Decision$ OR Identif$ OR Prognos$).tw. | 1201259 |
| 4 | (Decision$).ti,ab AND (Model$ OR Clinical$ OR Logistic Models).tw. | 57982 |
| 5 | (Prognostic).ti,ab AND (History OR Variable$ OR Criteria OR Scor$ OR Characteristic$ OR Finding$ OR Factor$ OR Model$).tw. | 86370 |
| 6 | *#1 OR #2 OR #3 OR #4 OR #5* | 1549027 |
| 7 | (Risk OR Multivariable OR Multivariate).tw. AND (Association OR Associated OR Biomarker OR Odds OR Marker).tw. | 374085 |
| 8 | *#6 or #7* | 1764950 |
| 9 | (2010).yr | 546698 |
| 10 | AIM.sb | 1,805,006 |
| **11** | **#8 AND #9** | **119,321** |
| **12** | **#10 AND #11** | **11,826** |
