# Supplementary material for: External validation of multivariable prediction models: a systematic review of methodological conduct and reporting
Source: BMC Med Res Methodol. 2014 Mar 19;14:40. doi: 10.1186/1471-2288-14-40 (PMC3999945; doi:10.1186/1471-2288-14-40)
Supplement: Additional file 2: Table S2 — Data Extraction Sheet. [file 1471-2288-14-40-S2.docx]

**Additional file 2: Table S2: Data Extraction Sheet**
