# Supplementary material for: External validation of multivariable prediction models: a systematic review of methodological conduct and reporting
Source: BMC Med Res Methodol. 2014 Mar 19;14:40. doi: 10.1186/1471-2288-14-40 (PMC3999945; doi:10.1186/1471-2288-14-40)
Supplement: Additional file 3: Table S3 — List of included studies. [file 1471-2288-14-40-S3.doc]

Additional file 3

Additional file 3: Table S3 List of included studies

A1. Abu-Assi E, Ferreira-Gonzalez I, Ribera A, Marsal JR, Cascant P, et al. (2010) “Do GRACE (Global Registry of Acute Coronary events) risk scores still maintain their performance for predicting mortality in the era of contemporary management of acute coronary syndromes?”. American Heart Journal 160: 826–834.

A2. Abu-Assi E, Gracia-Acuna JM, Ferreira-Gonzalez I, Pena-Gil C, Gayoso-Diz P, et al. (2010) Evaluating the Performance of the Can Rapid Risk Stratification of Unstable Angina Patients Suppress Adverse Outcomes With Early Implementation of the ACC/AHA Guidelines (CRUSADE) bleeding score in a contemporary Spanish cohort of patients with non-ST-segment elevation acute myocardial infarction. Circulation 121: 2419–2426.

A3. Airaksinen KE, Suurmunne H, Porela P, Niemela M, Vikman S, et al. (2010) Usefulness of outpatient bleeding risk index to predict bleeding complications in patients with long-term oral anticoagulation undergoing coronary stenting. American Journal of Cardiology 106: 175–179.

A4. Asimos AW, Johnson AM, Rosamond WD, Price MF, Rose KM, et al. (2010) A multicenter evaluation of the ABCD2 score's accuracy for predicting early ischemic stroke in admitted patients with transient ischemic attack. Annals of Emergency Medicine 55: 201–210.

A5. Ay C, Dunkler D, Marosi C, Chiriac AL, Vormittag R, et al. (2010) Prediction of venous thromboembolism in cancer patients. Blood 116: 5377–5382.

A6. Ay H, Gungor L, Arsava EM, Rosand J, Vangel M, et al. (2010) A score to predict early risk of recurrence after ischemic stroke. Neurology 74: 128–135.

A7. Bahl V, Hu HM, Henke PK, Wakefield TW, Campbell DA, Jr., et al. (2010) A validation study of a retrospective venous thromboembolism risk scoring method. Annals of Surgery 251: 344–350.

A8. Blankenberg S, Zeller T, Saarela O, Havulinna AS, Kee F, et al. (2010) Contribution of 30 biomarkers to 10-year cardiovascular risk estimation in 2 population cohorts: the MONICA, risk, genetics, archiving, and monograph (MORGAM) biomarker project. Circulation 121: 2388–2397.

A9. Bosner S, Haasenritter J, Becker A, Karatolios K, Vaucher P, et al. (2010) Ruling out coronary artery disease in primary care: development and validation of a simple prediction rule. CMAJ Canadian Medical Association Journal 182: 1295–1300.

A10. Brunelli A, Varela G, Refai M, Jimenez MF, Pompili C, et al. (2010) A scoring system to predict the risk of prolonged air leak after lobectomy. Annals of Thoracic Surgery 90: 204–209.

A11. Cadili A, Dabbs K, Scolyer RA, Brown PT, Thompson JF (2010) Re-evaluation of a scoring system to predict nonsentinel-node metastasis and prognosis in melanoma patients. Journal of the American College of Surgeons 211: 522–525.

A12. Chien KL, Lin HJ, Lee BC, Hsu HC, Lee YT, et al. (2010) A prediction model for the risk of incident chronic kidney disease. American Journal of Medicine 123: 836–846.

A13. Cima RR, Hassan I, Poola VP, Larson DW, Dozois EJ, et al. (2010) Failure of institutionally derived predictive models of conversion in laparoscopic colorectal surgery to predict conversion outcomes in an independent data set of 998 laparoscopic colorectal pedures. Annals of Surgery 251: 652–658.

A14. Collins GS, Altman DG (2010) An independent and external validation of QRISK2 cardiovascular disease risk score: a prospective open cohort study. BMJ 340: c2442.

A15. Cotton BA, Dossett LA, Haut ER, Shafi S, Nunez TC, et al. (2010) Multicenter validation of a simplified score to predict massive transfusion in trauma. Journal of Trauma-Injury Infection & Critical Care 69: Suppl-9.

A16. Craig JC, Williams GJ, Jones M, Codarini M, Macaskill P, et al. (2010) The accuracy of clinical symptoms and signs for the diagnosis of serious bacterial infection in young febrile children: prospective cohort study of 15 781 febrile illnesses. BMJ 340: c1594.

A17. Dubos F, Korczowski B, Aygun DA, Martinot A, Prat C, et al. (2010) Distinguishing between bacterial and aseptic meningitis in children: European comparison of two clinical decision rules. Archives of Disease in Childhood 95: 963–967.

A18. Durga P, Sahu BP, Mantha S, Ramachandran G (2010) Development and validation of predictors of respiratory insufficiency and mortality scores: simple bedside additive scores for prediction of ventilation and in-hospital mortality in acute cervical spine injury. Anesthesia & Analgesia 110: 134–140.

A19. Eastridge BJ, Butler F, Wade CE, Holcomb JB, Salinas J, et al. (2010) Field triage score (FTS) in battlefield casualties: validation of a novel triage technique in a combat environment. American Journal of Surgery 200: 724–727.

A20. El-Solh AA, Alhajhusain A, Abou JP, Drinka P (2010) Validity of severity scores in hospitalized patients with nursing home-acquired pneumonia. Chest 138: 1371–1376.

A21. Forest VI, Clark JJ, Veness MJ, Milross C (2010) N1S3: a revised staging system for head and neck cutaneous squamous cell carcinoma with lymph node metastases: results of 2 Australian Cancer Centers. Cancer 116: 1298–1304.

A22. Forno E, Fuhlbrigge A, Soto-Quiros ME, Avila L, Raby BA, et al. (2010) Risk factors and predictive clinical scores for asthma exacerbations in childhood. Chest 138: 1156–1165.

A23. Fueglistaler P, Amsler F, Schuepp M, Fueglistaler-Montali I, Attenberger C, et al. (2010) Prognostic value of Sequential Organ Failure Assessment and Simplified Acute Physiology II Score compared with trauma scores in the outcome of multiple-trauma patients. American Journal of Surgery 200: 204–214.

A24. Galetto-Lacour A, Zamora SA, Andreola B, Bressan S, Lacroix L, et al. (2010) Validation of a laboratory risk index score for the identification of severe bacterial infection in children with fever without source. Archives of Disease in Childhood 95: 968–973.

A25. Gaur P, Sepesi B, Hofstetter WL, Correa AM, Bhutani MS, et al. (2010) A clinical nomogram predicting pathologic lymph node involvement in esophageal cancer patients. Annals of Surgery 252: 611–617.

A26. Geisler CH, Kolstad A, Laurell A, Raty R, Jerkeman M, et al. (2010) The Mantle Cell Lymphoma International Prognostic Index (MIPI) is superior to the International Prognostic Index (IPI) in predicting survival following intensive first-line immunochemotherapy and autologous stem cell transplantation (ASCT). Blood 115: 1530–1533.

A27. Gondrie MJ, Mali WP, Jacobs PC, Oen AL, van der Graaf Y, et al. (2010) Cardiovascular disease: prediction with ancillary aortic findings on chest CT scans in routine practice. Radiology 257: 549–559.

A28. Grotenhuis BA, van HP, Reitsma JB, Lagarde SM, Wijnhoven BP, et al. (2010) Validation of a nomogram predicting complications after esophagectomy for cancer. Annals of Thoracic Surgery 90: 920–925.

A29. Gupta S, Berry JD, Ayers CR, Matulevicius SA, Peshock RM, et al. (2010) Association of Health Aging and Body Composition (ABC) Heart Failure score with cardiac structural and functional abnormalities in young individuals. American Heart Journal 159: 817–824.

A30. Hippisley-Cox J, Coupland C (2010) Individualising the risks of statins in men and women in England and Wales: population-based cohort study. Heart 96: 939–947.

A31. Hirose J, Ide J, Yakushiji T, Abe Y, Nishida K, et al. (2010) Prediction of postoperative ambulatory status 1 year after hip fracture surgery. Archives of Physical Medicine & Rehabilitation 91: 67–72.

A32. Hsu CY, Hsia CY, Huang YH, Su CW, Lin HC, et al. (2010) Selecting an optimal staging system for hepatocellular carcinoma: comparison of 5 currently used prognostic models. Cancer 116: 3006–3014.

A33. Jeldres C, Sun M, Lughezzani G, Isbarn H, Shariat SF, et al. (2010) Highly predictive survival nomogram after upper urinary tract urothelial carcinoma. Cancer 116: 3774–3784.

A34. Jimenez D, Aujesky D, Moores L, Gomez V, Lobo JL, et al. (2010) Simplification of the pulmonary embolism severity index for prognostication in patients with acute symptomatic pulmonary embolism. Archives of Internal Medicine 170: 1383–1389.

A35. Klatte T, Remzi M, Zigeuner RE, Mannweiler S, Said JW, et al. (2010) Development and external validation of a nomogram predicting disease specific survival after nephrectomy for papillary renal cell carcinoma. Journal of Urology 184: 53–58.

A36. Komatsu I, Tokuda Y, Shimada G, Jacobs JL, Onodera H (2010) Development of a simple model for predicting need for surgery in patients who initially undergo conservative management for adhesive small bowel obstruction. American Journal of Surgery 200: 215–223.

A37. Koton S, Bornstein NM, Tsabari R, Tanne D, Nasis I (2010) Derivation and validation of the prolonged length of stay score in acute stroke patients. Neurology 74: 1511–1516.

A38. Krug U, Rollig C, Koschmieder A, Heinecke A, Sauerland MC, et al. (2010) Complete remission and early death after intensive chemotherapy in patients aged 60 years or older with acute myeloid leukaemia: a web-based application for prediction of outcomes. Lancet 376: 2000–2008.

A39. Kruse RL, Parker OD, Mehr DR, Petroski GF, Swenson DL, et al. (2010) Using mortality risk scores for long-term prognosis of nursing home residents: caution is recommended. Journals of Gerontology Series A-Biological Sciences & Medical Sciences 65: 1235–1241.

A40. Kwak MS, Kim N, Lee HS, Lee HE, Jung HC, et al. (2010) Predictive power of serum pepsinogen tests for the development of gastric cancer in comparison to the histologic risk index. Digestive Diseases & Sciences 55: 2275–2282.

A41. Lappen JR, Keene M, Lore M, Grobman WA, Gossett DR (2010) Existing models fail to predict sepsis in an obstetric population with intrauterine infection. American Journal of Obstetrics & Gynecology 203: 573–575.

A42. Le Tourneau T, Pellikka PA, Brown ML, Malouf JF, Mahoney DW, et al. (2010) Clinical outcome of asymptomatic severe aortic stenosis with medical and surgical management: importance of STS score at diagnosis. Annals of Thoracic Surgery 90: 1876–1883.

A43. Leung YY, Tam LS, Ho KW, Lau WM, Li TK, et al. (2010) Evaluation of the CASPAR criteria for psoriatic arthritis in the Chinese population. Rheumatology 49: 112–115.

A44. Licurse A, Kim MC, Dziura J, Forman HP, Formica RN, et al. (2010) Renal ultrasonography in the evaluation of acute kidney injury: developing a risk stratification framework. Archives of Internal Medicine 170: 1900–1907.

A45. Maluenda G, Delhaye C, Gaglia MA, Jr., Ben-Dor I, Gonzalez MA, et al. (2010) A novel percutaneous coronary intervention risk score to predict one-year mortality. American Journal of Cardiology 106: 641–645.

A46. Manktelow BN, Draper ES, Field DJ (2010) Predicting neonatal mortality among very preterm infants: a comparison of three versions of the CRIB score. Archives of Disease in Childhood Fetal & Neonatal Edition 95: F9-F13.

A47. Matthews JC, Pagani FD, Haft JW, Koelling TM, Naftel DC, et al. (2010) Model for end-stage liver disease score predicts left ventricular assist device operative transfusion requirements, morbidity, and mortality. Circulation 121: 214–220.

A48. Miceli A, Duggan SM, Capoun R, Romeo F, Caputo M, et al. (2010) A clinical score to predict the need for intraaortic balloon pump in patients undergoing coronary artery bypass grafting. Annals of Thoracic Surgery 90: 522–526.

A49. Mitchell L, Lambers M, Flege S, Kenet G, Li-Thiao-Te V, et al. (2010) Validation of a predictive model for identifying an increased risk for thromboembolism in children with acute lymphoblastic leukemia: results of a multicenter cohort study. Blood 115: 4999–5004.

A50. Mitchell SL, Miller SC, Teno JM, Kiely DK, Davis RB, et al. (2010) Prediction of 6-month survival of nursing home residents with advanced dementia using ADEPT vs hospice eligibility guidelines. JAMA 304: 1929–1935.

A51. Moghaddam Y, Falzon M, Fulford L, Williams NR, Keshtgar MR (2010) Comparison of three mathematical models for predicting the risk of additional axillary nodal metastases after positive sentinel lymph node biopsy in early breast cancer. British Journal of Surgery 97: 1646–1652.

A52. Moore RG, Jabre-Raughley M, Brown AK, Robison KM, Miller MC, et al. (2010) Comparison of a novel multiple marker assay vs the Risk of Malignancy Index for the prediction of epithelial ovarian cancer in patients with a pelvic mass. American Journal of Obstetrics & Gynecology 203: 228–226.

A53. Nguyen CT, Yu C, Moussa A, Kattan MW, Jones JS (2010) Performance of prostate cancer prevention trial risk calculator in a contemporary cohort screened for prostate cancer and diagnosed by extended prostate biopsy. Journal of Urology 183: 529–533.

A54. O’Connor CM, Hasselblad V, Mehta RH, Tasissa G, Califf RM, et al. (2010) Triage after hospitalization with advanced heart failure: the ESCAPE (Evaluation Study of Congestive Heart Failure and Pulmonary Artery Catheterization Effectiveness) risk model and discharge score. Journal of the American College of Cardiology 55: 872–878.

A55. Onder G, Petrovic M, Tangiisuran B, Meinardi MC, Markito-Notenboom WP, et al. (2010) Development and validation of a score to assess risk of adverse drug reactions among in-hospital patients 65 years or older: the GerontoNet ADR risk score. Archives of Internal Medicine 170: 1142–1148.

A56. Peterson ED, Dai D, DeLong ER, Brennan JM, Singh M, et al. (2010) Contemporary mortality risk prediction for percutaneous coronary intervention: results from 588,398 procedures in the National Cardiovascular Data Registry. Journal of the American College of Cardiology 55: 1923–1932.

A57. Rades D, Douglas S, Veninga T, Stalpers LJ, Hoskin PJ, et al. (2010) Validation and simplification of a score predicting survival in patients irradiated for metastatic spinal cord compression. Cancer 116: 3670–3673.

A58. Rahmanian PB, Adams DH, Castillo JG, Carpentier A, Filsoufi F (2010) Predicting hospital mortality and analysis of long-term survival after major noncardiac complications in cardiac surgery patients. Annals of Thoracic Surgery 90: 1221–1229.

A59. Reed MJ, Newby DE, Coull AJ, Prescott RJ, Jacques KG, et al. (2010) The ROSE (risk stratification of syncope in the emergency department) study. Journal of the American College of Cardiology 55: 713–721.

A60. Regenbogen SE, Bordeianou L, Hutter MM, Gawande AA (2010) The intraoperative Surgical Apgar Score predicts postdischarge complications after colon and rectal resection. Surgery 148: 559–566.

A61. Rhatigan E, Tyrmpas I, Murray G, Plevris JN (2010) Scoring system to identify patients at high risk of oesophageal cancer. British Journal of Surgery 97: 1831–1837.

A62. Sartorius D, Le Manach Y, David JS, Rancurel E, Smail N, et al. (2010) Mechanism, glasgow coma scale, age, and arterial pressure (MGAP): a new simple prehospital triage score to predict mortality in trauma patients. Critical Care Medicine 38: 831–837.

A63. Schnabel RB, Aspelund T, Li G, Sullivan LM, Suchy-Dicey A, et al. (2010) Validation of an atrial fibrillation risk algorithm in whites and African Americans. Archives of Internal Medicine 170: 1909–1917.

A64. Seamon MJ, Franco MJ, Stawicki SP, Smith BP, Kulp H, et al. (2010) Do chronic liver disease scoring systems predict outcomes in trauma patients with liver disease? A comparison of MELD and CTP. Journal of Trauma-Injury Infection & Critical Care 69: 568–573.

A65. Sessler DI, Sigl JC, Manberg PJ, Kelley SD, Schubert A, et al. (2010) Broadly applicable risk stratification system for predicting duration of hospitalization and mortality. Anesthesiology 113: 1026–1037.

A66. Shamash J, Stebbing J, Sweeney C, Sonpavde G, Harland S, et al. (2010) A validated prognostic index predicting response to dexamethasone and diethylstilbestrol in castrate-resistant prostate cancer. Cancer 116: 3595–3602.

A67. Son JK, Lillehei CW, Gauvreau K, Jenkins KJ (2010) A risk adjustment method for newborns undergoing noncardiac surgery. Annals of Surgery 251: 754–758.

A68. Stiell IG, Clement CM, O’Connor A, Davies B, Leclair C, et al. (2010) Multicentre prospective validation of use of the Canadian C-Spine Rule by triage nurses in the emergency department. CMAJ Canadian Medical Association Journal 182: 1173–1179.

A69. Taylor CM, Humphries KH, Pu A, Ghali W, Gao M, et al. (2010) A proposed clinical model for efficient utilization of invasive coronary angiography. American Journal of Cardiology 106: 457–462.

A70. Thiruganasambandamoorthy V, Hess EP, Alreesi A, Perry JJ, Wells GA, et al. (2010) External validation of the San Francisco Syncope Rule in the Canadian setting. Annals of Emergency Medicine 55: 464–472.

A71. Tiedemann A, Lord SR, Sherrington C (2010) The development and validation of a brief performance-based fall risk assessment tool for use in primary care. Journals of Gerontology Series A-Biological Sciences & Medical Sciences 65: 896–903.

A72. Tsivgoulis G, Stamboulis E, Sharma VK, Heliopoulos I, Voumvourakis K, et al. (2010) Multicenter external validation of the ABCD2 score in triaging TIA patients. Neurology 74: 1351–1357.

A73. van den Hoven I, Kuijt GP, Voogd AC, van Beek MW, Roumen RM (2010) Value of Memorial Sloan-Kettering Cancer Center nomogram in clinical decision making for sentinel lymph node-positive breast cancer. British Journal of Surgery 97: 1653–1658.

A74. Vickers AJ, Cronin AM, Aus G, Pihl CG, Becker C, et al. (2010) Impact of recent screening on predicting the outcome of prostate cancer biopsy in men with elevated prostate-specific antigen: data from the European Randomized Study of Prostate Cancer Screening in Gothenburg, Sweden. Cancer 116: 2612–2620.

A75. Weisenthal BM, Chang AM, Walsh KM, Collin MJ, Shofer FS, et al. (2010) Relation between thrombolysis in myocardial infarction risk score and one-year outcomes for patients presenting at the emergency department with potential acute coronary syndrome. American Journal of Cardiology 105: 441–444.

A76. Wykrzykowska JJ, Garg S, Girasis C, de VT, Morel MA, et al. (2010) Value of the SYNTAX score for risk assessment in the all-comers population of the randomized multicenter LEADERS (Limus Eluted from A Durable versus ERodable Stent coating) trial. Journal of the American College of Cardiology 56: 272–277.

A77. Wysokinski WE, Ammash N, Sobande F, Kalsi H, Hodge D, et al. (2010) Predicting left atrial thrombi in atrial fibrillation. American Heart Journal 159: 665–671.

A78. Yu JB, Makarov DV, Sharma R, Peschel RE, Partin AW, et al. (2010) Validation of the partin nomogram for prostate cancer in a national sample. Journal of Urology 183: 105–111.
